# Probing Structure-Function Relationships in Missense Variants in the Carboxy-Terminal Region of BRCA1

**DOI:** 10.1371/journal.pone.0097766

**Published:** 2014-05-20

**Authors:** Renato S. Carvalho, Renata B. V. Abreu, Aneliya Velkova, Sylvia Marsillac, Renato S. Rodarte, Guilherme Suarez-Kurtz, Edwin S. Iversen, Alvaro N. A. Monteiro, Marcelo A. Carvalho

**Affiliations:** 1 Cancer Epidemiology Program, H. Lee Moffitt Cancer Center & Research Institute, Tampa, Florida, United States of America; 2 Programa de Farmacologia, Instituto Nacional de Câncer, Rio de Janeiro, RJ, Brazil; 3 Universidade Federal de Alagoas, Maceió, AL, Brazil; 4 Department of Statistical Science, Duke University, Durham, North Carolina, United States of America; 5 Instituto Federal de Educação, Ciência e Tecnologia do Rio de Janeiro, Rio de Janeiro, RJ, Brazil; CNR, Italy

## Abstract

Germline inactivating variants in *BRCA1* lead to a significantly increased risk of breast and ovarian cancers in carriers. While the functional effect of many variants can be inferred from the DNA sequence, determining the effect of missense variants present a significant challenge. A series of biochemical and cell biological assays have been successfully used to explore the impact of these variants on the function of BRCA1, which contribute to assessing their likelihood of pathogenicity. It has been determined that variants that co-localize with structural or functional motifs are more likely to disrupt the stability and function of BRCA1. Here we assess the functional impact of 37 variants chosen to probe the functional impact of variants in phosphorylation sites and in the BRCT domains. In addition, we perform a meta-analysis of 170 unique variants tested by the transcription activation assays in the carboxy-terminal domain of BRCA1 using a recently developed computation model to provide assessment for functional impact and their likelihood of pathogenicity.

## Introduction

Inherited *BRCA1* inactivating mutations are major determinants of breast and ovarian cancer risk, accounting for 46–68% of cases with a family history of breast cancer cases [1], [2], [3], [4]. Since 1996 genetic testing to identify mutations in *BRCA1* and *BRCA2* has been offered to women with a family history of breast and ovarian cancer [5], [6]. Presently several different assay platforms are used to investigate *BRCA* alterations, including amplicon-based Sanger sequencing, target capture followed by next-generation sequencing, and methods to detect large genomic rearrangements [5], [7], [8]. Variations found during sequencing include nonsense, frameshift, missense, splicing, and small insertions and deletions. Variants in *BRCA1* that lead to functional inactivation, either by compromising gene expression, correct splicing, or protein structure and stability are associated with an increased risk for cancer [9]. In many instances, inactivation can be inferred from the DNA sequence alone (*e.g.* nonsense or frameshift changes). However, in cases such as missense or splicing variants the resulting impact on function cannot be directly inferred. While many variants have been evaluated using functional assays and multifactorial statistical models [10], [11], cancer association has not been determined for several variants, referred to as Variants of Uncertain Clinical Significance (VUS).

An array of functional tests and computation prediction tools have been developed to aid in the determination of the functional impact of sequence variants of BRCA1, in particular, *in vitro* assays that assess the integrity and functionality of the N-terminal RING finger and the C-terminal BRCT tandem domains (tBRCT) of BRCA1 [11], [12]. Variants in these domains are more likely to have a functional impact [13], [14]. Analysis of these variants fulfills a double purpose: they provide information to aid in the classification of variants, and inform the biology of BRCA1 by pinpointing specific regions on the protein critical for different biochemical activities.

In this report we conduct an analysis of a large series of variants located in the carboxy-terminal domain of BRCA1 with a focus on a critical structural feature that is thought to stabilize the tandem BRCT domains and phosphorylation motifs. We used the transcription activation (TA) assay to analyze a total of 37 variants. These include 24 naturally-occurring VUS and 13 artificial variants to comprehensively probe phosphorylation sites and explore salt-bridge interactions present in the tandem BRCT, connecting the arginine residue at position 1699 and the glutamic acid residue at position 1836 [15], [16]. The TA assay has been extensively validated showing 100% sensitivity (0.73 to 1.0; 95%CI) and 88.9% specificity (0.52 to 0.99; 95%CI) using a reference dataset of variants classified by multifactorial models [17]. Finally, we conduct a combined meta-analysis of published transcription-based assays using a Bayesian statistical model, called VarCall [18], to assess the likelihood of pathogenicity given their functional impact.

## Materials and Methods

### Rationale for Choice of Variants

**Figure 1:**
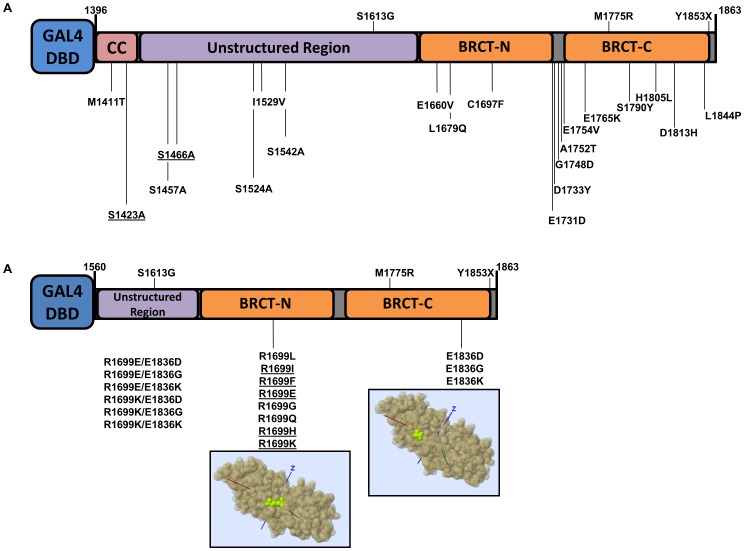
BRCA1 carboxy-terminal variants. *(* ***A*** *)* Natural and artificial (underlined) BRCA1 variants in the context of the analyzed region (comprising amino acids residues 1396–1863). *(* ***B*** *)* BRCA1 R1699 and E1836 variants in the context of the analyzed region (comprising amino acids residues 1560–1863). Structural models were obtained with Mupit tool (http://mupit.icm.jhu.edu/) using the 1jnx PDB structure (amino acid residues R1699 and E1936 are depicted in green).

In total we analyzed thirty seven *BRCA1* missense variants (Table 1, Figure 1). These variants represent three distinct groups: variants of uncertain significance in BRCA1, phosphorylation site variants, and salt-bridge variants in the BRCT domains. With the exception of R1699W, no other variant was found in the NHBLI Exome Sequencing Project (data release ESP6500 SI-V2).

**Table 1:** BRCA1 variants analyzed in this study.

Exon	Protein Variant (HGVS)	DNA variant (HGVS)	DNA variant (BIC)	Notes
13	p.Met1411Thr	c.4232T>C	T4351C	VUS; Coiled-coil domain
13	p.Ser1423Ala	c.4268G>A	G4386A	Disordered region; Phosphorylation site; artificial variant
14	p.Ser1457Ala	c.4369T>G	T4488G	Disordered region; Phosphorylation site
14	p.Ser1466Ala	c.4398C>A	C4517A	Disordered region; Phosphorylation site; artificial variant
15	p.Ser1524Ala	c.4570T>G	T4689G	Disordered region; Phosphorylation site
15	p.Ile1529Val	c.4585A>G	A4704G	Disordered region
15	p.Ser1542Ala	c.4624T>G	T4743G	Disordered region; Phosphorylation site
16	p.Glu1660Val	c.4979A>T	A5098T	BRCT;
17	p.Leu1679Gln	c.5036T>A	T5155A	BRCT
18	p.Cys1697Phe	c.5090G>T	G5209T	BRCT
19	p.Glu1731Asp	c.5193G>C	G5312C	BRCT
20	p.Asp1733Tyr	c.5197G>T	G5316T	BRCT
20	p.Gly1748Asp	c.5243G>A	G5362A	BRCT
20	p.Ala1752Thr	c.5254G>A	G5373A	BRCT
20	p.Glu1754Val	c.5261A>T	A5380T	BRCT
21	p.Glu1765Lys	c.5293G>A	G5412A	BRCT
22	p.Ser1790Tyr	c.5369C>A	C5488A	BRCT
23	p.His1805Leu	c.5414A>T	A5533T	BRCT
23	p.Asp1813His	c.5437G>C	G5556C	BRCT
24	p.Leu1844Pro	c.5531T>C	T5650C	BRCT
18	p.Arg1699Gly	c.5095C>G	5214	Salt bridge variants
18	p.Arg1699Leu	c.5096G>T	5215	Salt bridge variants
18	p.Arg1699Gln	c.5096G>A	5215	Salt bridge variants
18	p.Arg1699Glu	N/A	N/A	Salt bridge variants
18	p. Arg1699Phe	N/A	N/A	Salt bridge variants
18	p. Arg1699His	N/A	N/A	Salt bridge variants
18	p.Arg1699Ile	N/A	N/A	Salt bridge variants
18	p.Arg1699Lys	N/A	N/A	Salt bridge variants
24	p.Glu1836Ly	c.5506G>A	5625	Salt bridge variants
24	p.Glu1836Gly	c.5507A>G	5626	Salt bridge variants
24	p.Glu1836Asp	c.5508G>C	5627	Salt bridge variants
18/24	p.Arg1699Lys			Salt bridge variants (double mutants)
	p.Glu1836Lys			
18/24	p. Arg1699Lys			Salt bridge variants (double mutants)
	p.Glu1836Gly			
18/24	p. Arg1699Lys			Salt bridge variants (double mutants)
	p.Glu1836Asp			
18/24	p.Arg1699Glu			Salt bridge variants (double mutants)
	p.Glu1836Lys			
18/24	p.Arg1699Glu			Salt bridge variants (double mutants)
	p.Glu1836Gly			
18/24	p.Arg1699Glu			Salt bridge variants (double mutants)
	p.Glu1836Asp			

Non-naturally occurring variants are underlined.

#### Variants of uncertain significance

Five variants (M1411T, S1457A, S1524A, I1529V and S1542A) were chosen to increase coverage of the unstructured region (aa 1396–1648) of BRCA1 C-terminus (aa 1396–1863, corresponding to exons 13 to 24 coding sequence, encompassing the two BRCT tandem domains) [19]. Three of these naturally-occurring variants (S1457A, S1524A and S1542A) are located in BRCA1 phosphorylation sites. Several phosphorylation sites have been identified on the C-terminal of BRCA1 that are involved in biological functions such as cell cycle checkpoint and caspase activation [20]. We also generated artificial (cannot result from a single nucleotide change in the natural codon) alanine variants to probe the role of phosphorylation at position S1423 and S1466 to be consistent with the other phosphorylation sites. Alanine was choosen to substitute serine residues in order to impede phosphorylation. The remaining 13 variants (E1660V, L1679Q, C1697F, E1731D, D1733Y, G1748D, A1752T, E1754V, E1765K, S1790Y, H1805L, D1813H and L1844P) are missense variants in the tBRCT domains (Table 1, Figure 1).

#### BRCT salt-bridge variants

In previous studies we identified and analyzed a naturally occurring *BRCA1* allele, R1699W, which showed a temperature-sensitive behavior [16], [21]. R1699 and E1836 are critical residues for a salt-bridge that stabilizes the tBRCT [15], [16]. We reasoned that variants in residues involved in the salt-bridge could generate useful structure-function information and potential temperature-sensitive proteins [21]. Thus, we generated a panel of eight variants of residue 1699 and three variants of residue 1836. We also generated four double mutants combining changes in residues R1699 and E1836 (Table 1, Figure 1).

### Constructs

BRCA1 expression constructs (controls and variants) were generated in 2 different construct contexts: for VUS and phosphorylation sites variants analysis we used the region comprising amino acid residues 1396–1863, corresponding to exon 13–24 coding sequence (Figure 1A); for salt-bridge variants analysis we used the region comprising amino acid residues 1560–1863, corresponding to exon 16–24 coding sequence (Figure 1B). All variants were confirmed by sequencing.

#### VUS and phosphorylation sites variants

Control constructs (amino acid context 1396–1863) containing the wt BRCA1, S1613G, M1775R, and Y1853X were generated previously (Figure 1A) [22]. Mutations in the cDNA sequence were introduced by site-directed mutagenesis using plasmid p385-BRCA1 as template, as previously described [23]. Primers sequences are available upon request. For each variant, both products (5′ and 3′ regions) were combined and used as a template for a final round of PCR using 24ENDT and UX13 primers [23]. The final PCR products were then digested with *Bam*H1 and *Eco*R1 and ligated to pGBT9 vector. To obtain the heterologous GAL4 DNA binding domain (GAL4-DBD) fusions in a mammalian expression vector, pGTB9 constructs were digested with *Hind*III and *Bam*H1, a 1.8 Kb band was isolated and ligated into equally digested pCDNA3 vector.

#### BRCT salt-bridge variants

Control constructs (amino acid context 1560–1863, Figure 1B) containing the wt BRCA1, M1775R, and Y1853X were previously described [16], [24]. Mutations in the cDNA sequence were introduced as described above. To obtain the double mutants the same procedure and primers described were used but instead of wild-type cDNA as templates we used the individual constructs containing the single mutants at position 1699. The final PCR products were then digested and cloned in pGBT9 vector, then subcloned in pCDNA3 as described above.

### Transcriptional Assay

The transcriptional assays were performed in mammalian cells as described [12], [19], [22]. Briefly, we used pG5Luc as a reporter and transfections were normalized with an internal control phGR-TK (Promega), which contains a *Renilla* luciferase gene under a constitutive TK basal promoter. Transfections were conducted in human HEK293T, HCC1937 [25] or SUM1315 cells in triplicate using Fugene 6 (Roche), harvested 24 h post-transfection, and luciferase activity was measured using the Dual Luciferase Reporter Assay System (Promega). Results were plotted as a percentage of the wild-type activity. We inferred disease relevance for each missense variant using a computational tool, VarCall, based on a Bayesian hierarchical model. [18].

VarCall is a tool that uses functional data, quantitative or categorical, as input and accounts for sources of experimental heterogeneity. Specifically, here we used non-normalized ratios of Firefly luciferase/Renilla luciferase indexed by batch as the specific input data and each batch also records the ratio for the wild type control. VarCall generates a likelihood of pathogenicity for each variant given the input data in the form of a posterior probability of being damaging. In addition, VarCall also generates a Bayesian integrated likelihood ratio statistic that measures the degree to which the data support the hypothesis that a variant is protein damaging. To generate accurate probabilities VarCall uses large datasets and re-assesses previously anazide variants given the new data. Therefore, we included data for all (n = 176) variants previously tested under controlled conditions (see Results for further details). We used this model to infer disease relevance for all the naturally occurring variants described in this study, an additional set of 119 variants previously assayed for transcriptional activation [17] and provide a reanalysis of the 82 variants previously assessed by this model [18]. This combined set includes 2,436 measurements of transcriptional activity for 176 unique variants in the region analyzed in the assay: amino acid residues context 1396–1863 (Tables S1 and S2). Single nucleotide changes in this region can generate 2,740 unique variants (1,244 in the tBRCT). Of those, 219 have been documented in the population (BIC Database as of this writing) with 129 unique variants in the tBRCT domains. The present dataset represents 61% and 89.9% coverage of all variants documented in the amino acid region limited by residues 1398–1863 and the tBRCT domains, respectively. VarCall can also assess results from different construct contexts (aa 1396–1863 or aa 1560–1863) because each variant is assessed relative to the wild type in the same context. We have extensively tested several contexts for the TA assay including aa 1560–1863 [26], aa 1396–1863 [22], and aa 1646–1859 [17]. While they show different absolute activities the relative activities of variants (for example M1775R) are comparable in terms of the percentage of activity of the corresponding wild type. Thus, data from different contexts can be directly compared using VarCall since each batch is compared against its own wild-type construct.

## Results

### BRCA1 C-terminus VUS

**Figure 2:**
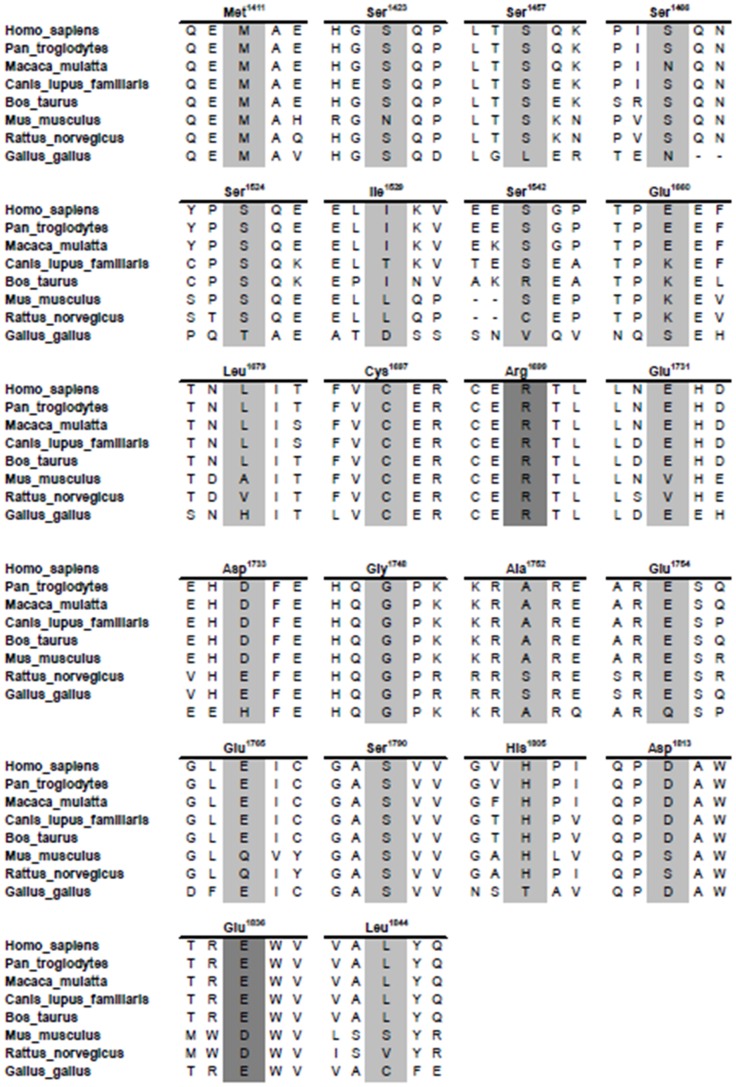
Phylogenetic conservation of amino acid residues in the surround region of BRCA1 analyzed variants. A multiple sequence alignment demonstrating amino acids residues evolutionary conservation are shown from *H.sapiens* (NP_009225.1), *P.troglodytes* (NP_001038958.1), *M. mulatta* (NP_001108421.1), *C.lupus* (NP_001013434.1), *B.taurus* (NP_848668.1), *M. musculus* (NP_033894.3), *R. norvegicus* (NP_036646.1) and *G. gallus* (NP_989500.1). Target amino acids residues are depicted in light grey and salt-bridge involved residues in dark grey.

In order to increase the coverage of variants located at carboxy-terminus of BRCA1 we selected a set of 18 naturally occuring missense variants (Table 1). Six are located outside the tandem BRCT (tBRCT) domains: M1411T, located in the coiled-coil region; S1457A, S1524A, I1529V, and S1542A, located in the unstructured region of BRCA; and L1844P located in the carboxy-terminal tail of the molecule. Twelve are located in tBRCT domains: E1660V, L1679Q, C1697F, E1731D, D1733Y, G1748D, A1752T, E1754V, E1765K, S1790Y, H1805L and D1813H (Figure 1A). To comprehensively probe the role of the phosphorylation sites we also included two artificial variants located outside the tBRCT that are ATM phosphorylation target sites: S1423A and S1466A [20] (Table 1, Figure 1A). All variants are located at amino acid residues strongly conserved in the vertebrate lineage (Figure 2).

**Figure 3:**
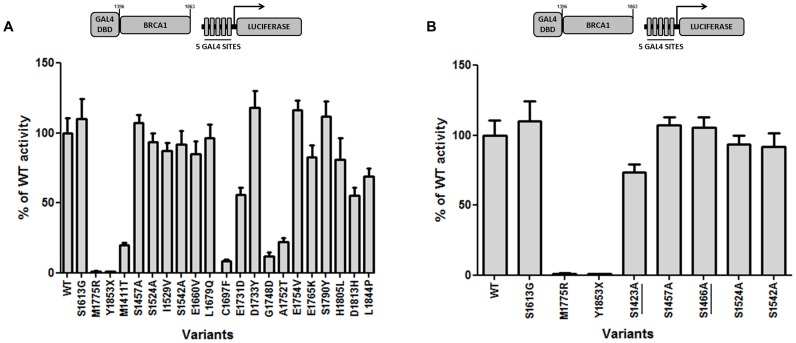
Functional analysis of missense variants in BRCA1 C-terminal region. Transcriptional activity of BRCA1 variants were evaluated in HEK293T cells using a GAL4-responsive firefly luciferase reporter gene (shown above the graphs) at 37°C. Cells were harvested 24h after transfections and the lysate was used to assess transcriptional activation ability by luciferase activity measurement. Activity is depicted as % of the wild-type activity. *(* ***A*** *)* Natural missense variants and *(* ***B*** *)* natural and artificial (underlined) variants located on phosphorylation sites. S1613G, M1775R and Y1853X variants were used as controls.

Among the naturally occuring variants, I1529V, E1660V, L1679Q, D1733Y, E1754V, E1765K, S1790Y, H1805L and L1844P showed transcriptional activity values corresponding to the wild-type reference (Figure 3A) indicating that they have no detectable functional impact. Similarly, the naturally-occurring phosphorylation variants S1457A, S1524A, and S1542A, and the artificial phosphorylation site variants, S1423A, S1466A, also displayed transcriptional activity >75% of the wild-type activity (Figure 3B) suggesting that phosphorylation at these sites is not required for the transcriptional activity of BRCA1 in normal growing conditions.

The remaining six variants exhibited reduced functional activity. Variants M1411T, C1697F, G1748D and A1752T showed significantly reduced (<30% of wt) levels of activity (Figure 3A). Two variants, E1731D and D1813H presented activty levels around 50% of the wild type reference (55±10% and 55±11% respectively, Figure 3A) suggesting that they might have a mild to moderate impact on the function of BRCA1.

### BRCT Salt-bridge Variants

The structural integrity of BRCA1 tBRCT is required for transcriptional activation activity and variants that disrupt its integrity are strongly correlated to cancer risk [17]. Previously we identified a naturally occurring *BRCA1* allele, R1699W, that displayed impaired transcriptional activity in standard conditions (37°C), but showed normal activity when cells are shifted to 30°C indicating that the protein is able to restore proper folding at lower temperature [21]. Further analysis also revealed a significant decrease in peptide binding sensitivity/specificity in comparison to wild-type [17]. Interestingly, the arginine residue at position 1699 is involved in a salt bridge with a glutamic acid and an aspartic acid at positions 1836 and 1840, respectively [15]. In order to dissect the role of these residues in this molecular interaction we generated a series of BRCA1 tBRCT single amino acid variants at positions 1699 and 1836 (Figure 1B).

**Figure 4:**
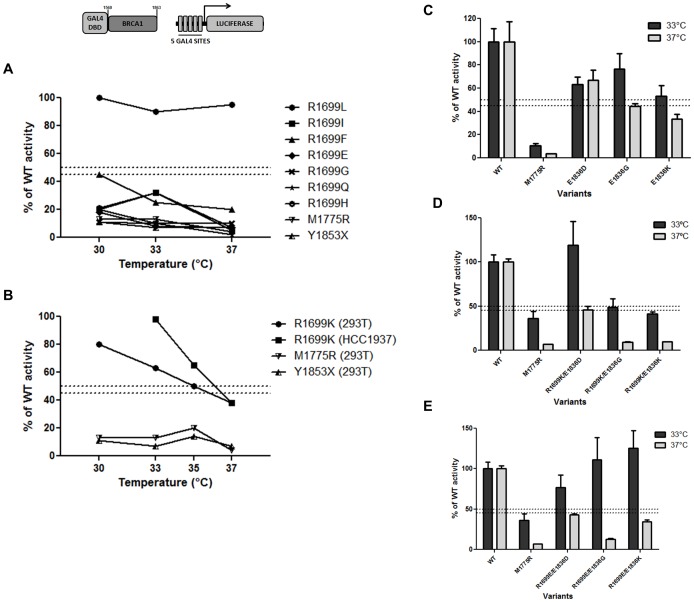
Functional analysis of BRCA1 R1699 and E1836 variants at different temperatures. Transcriptional activity of BRCA1 variants were evaluated using a GAL4-responsive firefly luciferase reporter gene (shown above the graphs). Cells were harvested 24h after transfections and the lysate was used to assess transcriptional activation ability by luciferase activity measurement. Activity is depicted as % of the wild-type activity. *(* ***A*** *)* Transcriptional activity of R1699L, R1699I, R1699F, R1699E, R1699G, R1699Q and R1699H variants and the controls M1775R and Y1853X at different temperatures evaluated in HEK293T cells; *(* ***B*** *)* transcriptional activity of the R1699K variant and the controls M1775R and Y1853X at different temperatures evaluated in HEK293T and HCC1937 cells and *(* ***C*** *)* transcriptional activity of the E1836D, E1836G and E1836K variants and the control M1775R at different temperatures evaluated in HEK293T. Transcriptional activity of double variants *(* ***D*** *)* R1699K/E1836D, R1699K/E1836G and R1699K/E1836K *(* ***E*** *)* R1699E/E1836D, R1699E/E1836G and R1699E/E1836K and the control M1775R at different temperatures evaluated in HEK293T at different temperatures.

We investigated these variants using the transcription activation assay in three different temperatures to assess temperature-sensitive behaviors (Figures 4A and 4B). Variants R1699I, R1699F, R1699E, R1699G, R1699Q and R1699H showed low activity across different temperatures. Interestingly, variant R1699L displayed an activity comparable to the wild type reference consistent across different temperatures (Figure 4A). One variant, a conservative change from an arginine residue to a lysine, R1699K, displayed a temperature dependent behavior, presenting transcriptional activation ∼80% of wt at 30°C and progressively decreasing with temperature. At 37°C this variant showed a significantly reduced activity (38% of wt, Figure 4B). This behavior was consistent in two different cell lines, HEK293T and HCC1937.

We also tested three variants at position 1836, where the original glutamic acid residue was replaced by an aspartic acid, a glycine or a lysine. E1836D exhibited a somewhat reduced transcriptional activation values that were comparable at 33°C and 37°C (Figure 4C). Variants E1836G and E1836K, on the other hand exhibited a temperature-sensitive behavior (Figure 4C).

Then, we combined the R1699K (temperature-sensitive) or the R1699E (not temperature-sensitive) with variations at the 1836 site and tested the double mutants at 33°C and 37°C. All R1699K double mutants retained the temperature-sensitive behavior but only the conservative E1836D mutations retained normal activity at the permissive temperature (33°C) highlighting the role of both residues (1699 and 1836) on the stability of the BRCT domains (Figure 4D). Moreover, changes in one residue can compensate for changes in the other as demonstrated by the R1699E double mutants, which show levels of activity comparable to wt at the permissive temperature but dramatically reduced levels when cells are shifted to 37°C. In particular, the R1699E/E1836G showed the largest difference of activity between the two temperatures and might constitute a useful tool to probe the function of BRCA1 (Figure 3E).

### VarCall

**Figure 5:**
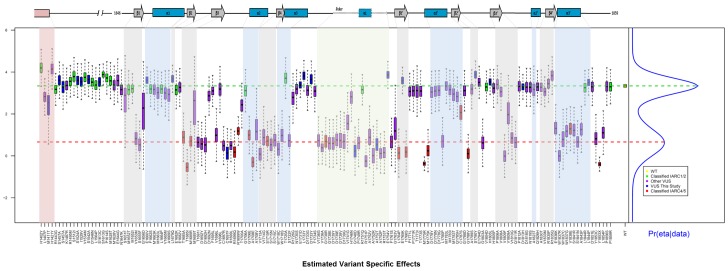
Estimated Variant Specific Effects (Bayesian statistical model, VarCall, graphical summary). Variants are depicted in order of amino acid from residues 1396 to 1863. The top panel shows secondary structures in the C-terminal region. Coiled-coil region and α-helixes are depicted as pink and blue boxes, respectively. β-sheets are shown as gray arrows. The shaded area on the graph corresponds to different structures of similar color. The linker region is indicated with green shading. Each variant’s activity is represented by a boxplot summarizing the marginal posterior distribution of its random effect. A point estimate of the mixture model is plotted on the right margin. Its top component corresponds to variants with no functional impact, whereas its bottom component corresponds to variants with functional impact. The mean of the benign/damaging component is plotted as a green/red dotted horizontal line. Yellow box represents wild-type reference, green and red boxes represent low (class 1 and 2) and high (class 4 and 5) risk variants respectively, classified according to the International Agency for Research on Cancer (IARC), purple boxes represent VUS previously analyzed by our group (used to feed VarClass algorithm), and blue boxes represent the VUS analyzed by the first time in this study.

Next, we evaluated the activity of missense variants using the VarCall computational model [18]. Results from this model reflect the likelihood of pathogenicity given the functional impact data. VarCall results are depicted in Figure 5 where each variant’s activity is represented by a boxplot summarizing the marginal posterior distribution of its random effect. A point estimate of the mixture model is plotted on the right margin. Its top component corresponds to variants with no functional impact, whereas its bottom component corresponds to variants with functional impact. VarCall data indicate that C1697F, G1748D and A1752T have significant impact on function and are likely to represent pathogenic variants (Figure 5). M1411T variant also showed reduced activity (Figure 5), resulting in a reduced but still significant probability of being pathogenic (0.35, Table S1).

## Discussion

A significant percentage of genetic tests conducted for breast and ovarian cancer susceptibility results in findings of a variant of uncertain clinical significance (VUS). While some VUS are located in intronic or other putative regulatory regions, many are missense variants. These individual VUS alleles usually are very rare in the population precluding family-based or population-based genetic analysis to determine their disease association. Functional assay have been used to determine whether specific amino acid changes lead to detectable functional impact in a number of biochemical and biological processes that have been associated with BRCA1 ([11] and references therein).

In this study we focused on the transcriptional activation assays for BRCA1. The method is based on the ectopic expression of BRCA1 C-terminal fragments fused to GAL4-DBD and the ability of the resulting chimeric protein to activate transcription of a reporter gene [27], [28]. Interestingly, there is a strong correlation of transcriptional activation results and other biochemical activities assigned to BRCA1 such as the specific recognition of phosphorylated peptides [17] indicating that the transcriptional assay functions as a monitor of the structural integrity of the BRCA1 C-terminal region. Importantly, the TA displays a strong correlation with pathogenicity indicating that the assay is specific and sensitive for BRCA1 missense variants in the C-terminus [17].

We analyzed 18 naturally occurring BRCA1 VUS (Figure 1A) using the TA assay [11], [19]. We also tested two artificial missense variants targeting phosphorylation sites in BRCA1 (Figure 1A). Transcriptional activity data was assessed by VarCall, a recently developed computational tool to infer disease association from functional data [18].

M1411T, C1697F, G1748D, and A1752T displayed significantly decreased transcriptional activity compared to the wt reference and were, with the exception of M1411T, determined to be likely to be pathogenic by VarCall (Figure 5, Table S1). Different variants on the same positions (A752V, A1752P, and C1697R) lead to severe protein folding defects inferred by increased protease sensitivity and impaired transcriptional activity [17]. Taken together these data highlight the relevance of these amino acid residues (C1697 and A1752) for the integrity of the BRCA1 tBRCT structure. Assessment of M1411T by VarCall was inconclusive. This variant, first reported in a Swedish ovarian cancer patient with a family history of breast cancer and other malignancies [29], lies on the BRCA1 coiled-coil domain and was reported to disrupt the interaction between BRCA1 and PALB2 [30].

All other natural variants tested (S1457A, S1524A, I1529V, S1542A, E1660V, L1679Q, E1731D, D1733Y, E1754V, E1765K, S1790Y, H1805L, D1813H and L1844P) displayed transcriptional activity comparable to the wt reference, as did the two artificial variants analyzed (S1423A and S1466A). These natural variants were determined to be unlikely to be pathogenic by VarClass (Figure 5, Table S1). Because unphosphorylatable (alanine) variants at phosphorylation sites did not have impact on transcriptional activity we conclude that phosphorylation of these residues is not required for transcription activation under normal conditions. They also provide a demonstration that serine to alanine substitutions in these residues do not induce dramatic structural changes to the tBRCT domains. Our data do not address the relevance of these variants following DNA damage, but it is clear that these sites are critical for DNA injury induced ATM phosphorylation of BRCA1, and the overall repair response [13], [20], [31], [32].

Next, we probed the role of the salt bridge that stabilizes the tandem BRCT interaction between residues R1699, E1836, and D1840 [15]. The temperature-dependent effects of the R1699W variant on transcriptional activity were previously reported [21]. We performed site-directed mutagenesis generating a series of eight variants of this residue (Figure 1B). Except for R1699L that behaved as reference in all tested conditions, and R1699K, discussed below, all other variants showed low TA values in all ranges of temperature tested (30°C to 37°C, Figure 4). Interestingly, the R1699L variant displayed impaired phosphopeptide binding activity [17] suggesting that this variant can be used to uncouple transcriptional activation from phosphopeptide binding. In addition to its role in the salt bridge, R1699L and R1699W, are predicted to abolish the interaction of the T1700 residue with PRKCD, PRKC, PRKCQ, PRKCZ, PRKCA, PRKCG and MST2 [33], [34]. Interestingly, R1699K was found to have a temperature-dependent behavior, showing about 80% of wild-type activity at 30°C and <40% activity at 37°C. These results were confirmed in the *BRCA1*-deficient HCC1937 cells [25] (Figure 4).

We also generated aspartic acid, glycine and lysine variants at position 1836 (Figure 1B). While the conservative change E1836D showed no temperature-dependent behavior, the E1836G and E1836K have significantly lower transcriptional activity at 37°C. E1836K was also reported to have modest effects on protein folding and transcriptional activation but significant decrease in peptide binding and specificity [17] in a pattern reminiscent of the R1699L variant. Then, we combined variants for R1699K and R1699E, which had temperature dependent and independent behaviors, respectively (Figure 1B and 4) with variants in E1836.

As expected, for double variants of R1699K, the substitution of a glutamic acid by an aspartic acid (E1836D) did not change the pattern observed for the single R1699K. R1699K double variants containing a non-charged (E1836G) or a positively charged amino acid residue (E1836K) resulted in reduced transcriptional activities even at low temperatures (Figure 4). R1699E double variants showed the largest difference in activities from the permissive to the non-permissive temperature of all variants, suggesting that these double variants can be used as genetic tools to investigate the role of different biochemical processes mediated by the BRCT domains.

The study of temperature influence on BRCA1 structure/function is especially relevant because temperature-sensitive variants may have different clinical presentations or penetrances. The evaluated variants could, judged by the behavior of the R1699W allele be potential intermediate risk variants. [21].

Finally, combined with the natural variants in this study (Table S1) we examined 170 unique variants using VarCall and generated likelihoods of pathogenicity. Note that because this tool uses a Bayesian approach every variant, even the 82 variants assessed previously [18] are re-evaluated given the new data. This analysis (Figure 5) reveals interesting insights about the architecture of the C-terminus. Some variants in the coiled-coil region displayed functional impact albeit a moderate one. The disordered region, located N-terminally to the BRCT domains is tolerant to changes. Similarly, α1 helixes on both BRCTs seem also to be tolerant to changes. Otherwise, most secondary structures and the linker region tend to be sensitive to changes. A note of caution is warranted here as these results, obtained in a research environment, are not meant to guide clinical decisions. Although the VarCall tool infers the likelihood of a variant being pathogenic, the results provided here are derived from a single data source (activity of a functional assay). Only determination of pathogenicity by a multifactorial likelihood model using independent data sources (*e.g.* segregation analysis, allele frequency, tumor pathology markers, co-occurrence, and co-observation with BRCA2 pathogenic variants) should be considered clinically [35], [36], [37].

In summary, in this paper we directly assessed the transcriptional assay of several BRCA1 VUS and conducted a comprehensive analysis of 170 unique variants using the VarCall computational tool. We also report data on several natural and artificial variants with temperature dependent behavior that can be utilized as reagents to dissect the functions of BRCA1.

## Supporting Information

Table S1 **Posterior probabilities and log Bayes Factor from VarCall.**
(XLSX)

Table S2 **Variant Dataset for VarCall.**
(XLSX)
